# The breast cancer that wasn't: Breast abscess mimicking malignancy

**DOI:** 10.1016/j.radcr.2024.09.120

**Published:** 2024-10-23

**Authors:** Bhavana Devanabanda, Ashani Shah, Mirette Shafeek, Magdalena Salvador

**Affiliations:** Rutgers New Jersey Medical School, 185 S Orange Ave, Newark, NJ 07103, USA

**Keywords:** Abscess, Mastitis, Inflammatory breast cancer

## Abstract

Mastitis, or inflammation of breast tissue, typically presents in lactating women but may be seen across all ages and in both genders. Infection is the most common etiology underlying this condition, although noninfectious inflammation is also possible. Timely diagnosis and treatment can prevent complications including breast abscess. Mastitis and breast abscess are both characterized by pain and may result in misdiagnosis and delayed treatment of more serious conditions such as inflammatory breast cancer (IBC). Conversely, imaging may result in misdiagnosis of breast abscesses due to the similarity to lesions suspicious for malignancy. We present the case of a 52-year-old female with imaging studies suspicious for IBC who was eventually diagnosed with breast abscess via pathological confirmation. We discuss the overlap and differences between breast abscess and breast malignancy regarding patient presentation, population, risk factors, and imaging findings for diagnostic purposes.

## Introduction

Breast abscesses and malignancies often share similar clinical and imaging features, which can complicate diagnosis. Both conditions may present as palpable masses with localized skin changes, and imaging can sometimes suggest malignancy. Breast cancer is the most common cancer among women worldwide, with a lifetime incidence of 13% in the United States [1]. Risk factors include age, family history, hormone use, and obesity. IBC is rare, accounting for about 2%-4% of cases, but it is particularly aggressive and contributes to approximately 7% of breast cancer mortality [2]. Consequently, a high degree of suspicion is essential when investigating breast changes, including masses.

Breast abscesses are categorized into puerperal and nonpuerperal types [3,4]. Nonpuerperal abscesses occur outside the breastfeeding period and are classified as either central (periareolar) or peripheral [4,5]. Risk factors for nonpuerperal abscesses include African American ethnicity, obesity, tobacco use, and diabetes, with some studies reporting diabetes in up to 64% of cases [6,7]. Radiologists play a pivotal role in the identification, characterization, and management of these conditions.

## Case presentation

**Fig. 1:**
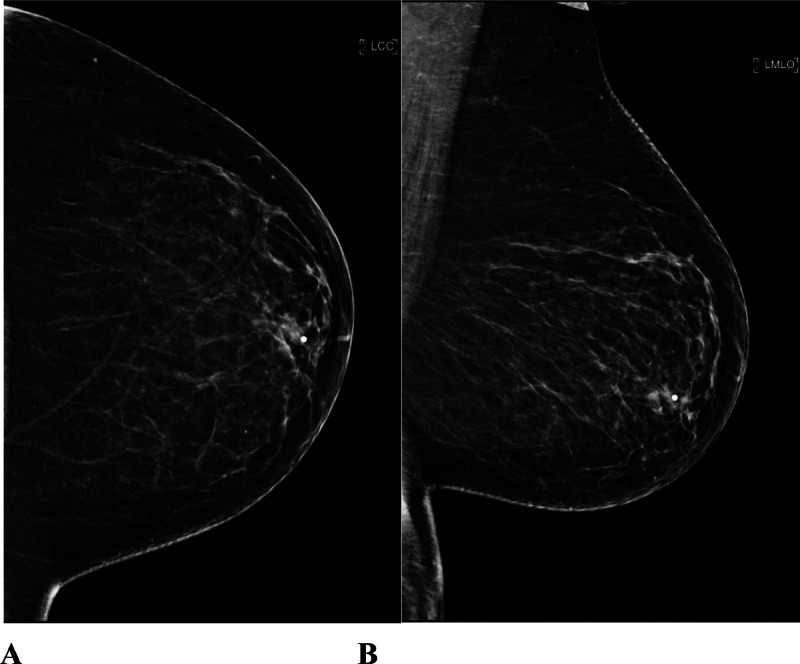
Screening mammogram of the left breast 1 year prior. (A) is a craniocaudal (CC) view and (B) is a mediolateral oblique (MLO) view. No suspicious masses, architectural distortion or suspicious calcifications in both views.

**Fig. 2:**
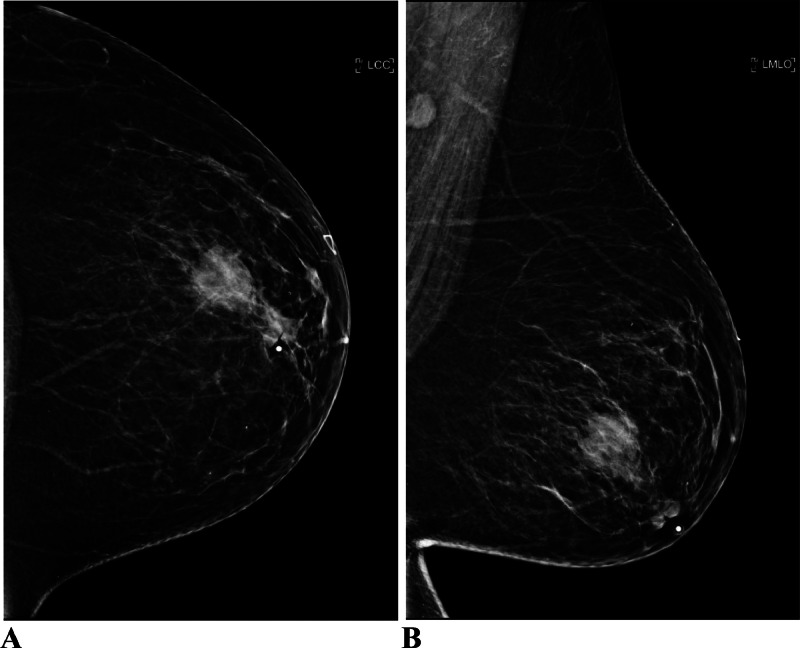
Most recent diagnostic mammogram of the left breast. (A) CC view and (B) MLO view. New retro-areolar mass measuring 2.5 cm with associated nipple retraction.

**Fig. 3:**
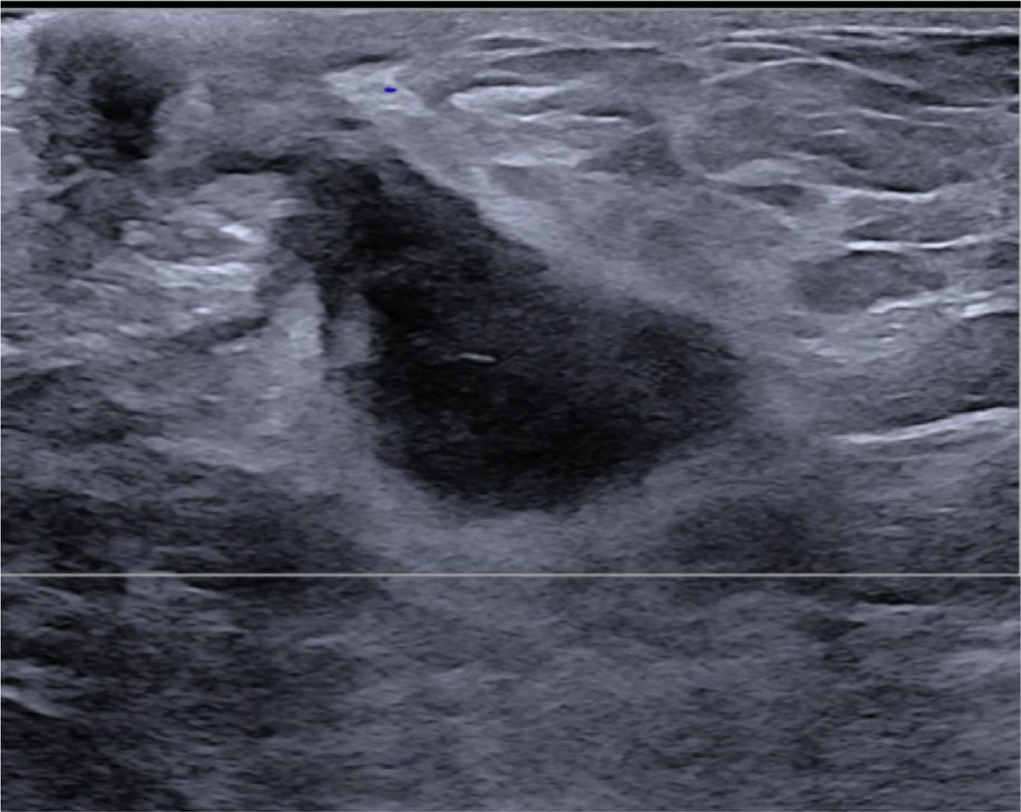
Retro-areolar left breast US: irregular complex cystic and solid mass.

A 52-year-old postmenopausal female with history of uncontrolled type II diabetes mellitus, neuropathy, hypertension, hyperlipidemia, morbid obesity and chronic smoker with 1 pack a day for the past 15 years initially presented to the outpatient Breast Clinic with a left breast swelling with associated nipple retraction. She had noticed the changes in her breast over the span of 4 weeks. She denied pain, erythema and tenderness. A screening mammography performed 1 year earlier was normal (Fig. 1). A diagnostic mammogram of the left breast showed a new left retro-areolar mass measuring 2.5 cm with associated nipple retraction (Fig. 2). Complete left breast ultrasound (US) showed an irregular complex cystic and solid mass measuring 2.7 × 2.3 × 1.3 cm at the 3:00 axis retro-areolar location (Fig. 3). The left axillary lymph nodes were normal in the United States. Findings were highly suggestive of malignancy. A US guided biopsy showed thick odorless non purulent brown fluid. Pathology from the breast biopsy showed lymphoplasmacytic infiltrate, histiocytes, neutrophils, multinucleated giant cells and fat necrosis consistent with abscess.

**Fig. 4:**
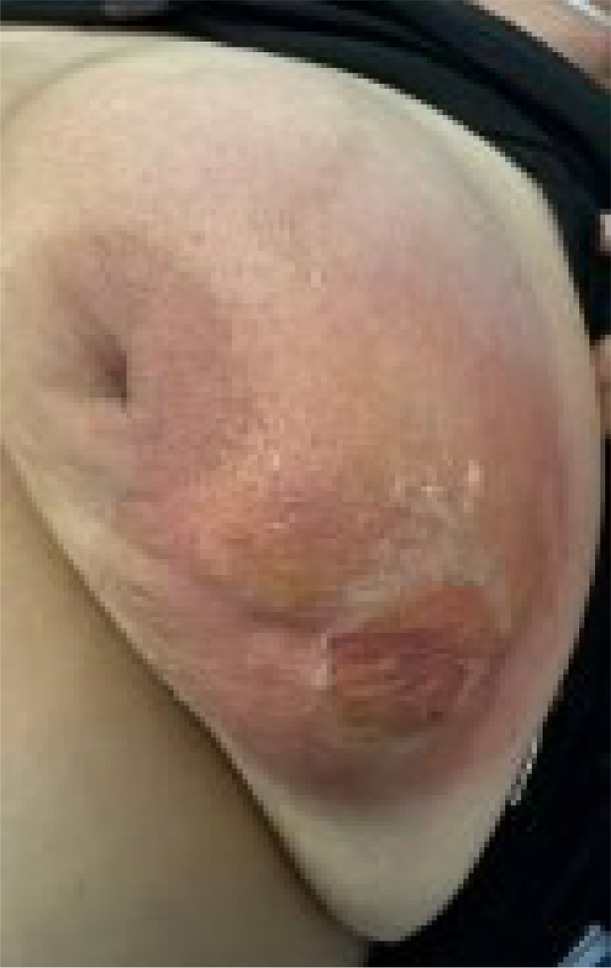
Left nipple retraction. Erythema and skin scaling on the left lateral breast.

Patient's primary care physician started her on oral cephalexin. Ten days after the biopsy, she presented to the emergency department with worsening tenderness, swelling, erythema and serosanguinous discharge at the site of the biopsy on the left lateral breast (Fig. 4). No crepitus or fluctuance was seen. Patient denied any fevers and chills. Patient denied family history of cancer. She had elevated white blood cell count 13 × 10*3/µL (4.0-11.0 × 10*3/µL). Elevated glucose of 165 mg/dl (70-109 mg/dl). Elevated c-reactive protein 16 mg/L (0-5 mg/L). Elevated erythrocyte sedimentation rate 40 mm/hour (0 - 20 mm/hour). HBA1C of 11.8% (4.8%-5.9%). Normal procalcitonin 0.14 (≤0.50 ng/mL). Blood pressure 137/87 mmHg, pulse 81 beats per minute, temperature 98°F (36.7°C), respiratory rate 20 breaths per minute, body mass index 43.3 kg/m2. She had developed acute cellulitis in the setting of a breast abscess.

**Fig. 5:**
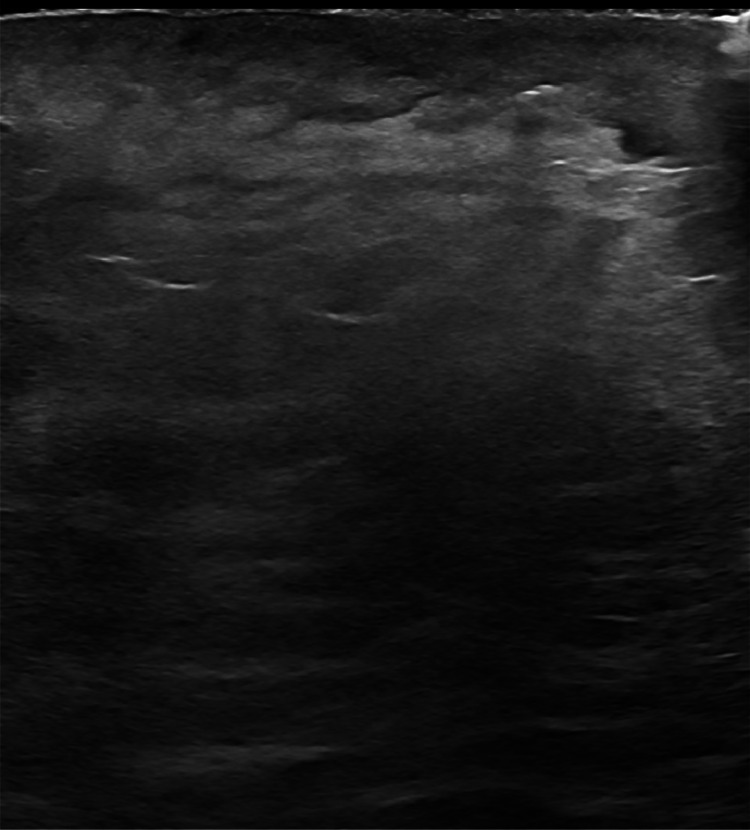
Left lateral breast demonstrates diffuse edema and skin thickening. Complex cyst is resolved.

Based on the antibiotic sensitivity panel, cephalexin was discontinued, and the patient was started on intravenous ceftriaxone and metronidazole. A repeat breast US was performed to evaluate for a drainable fluid collection. Targeted US of the lateral left breast showed diffuse swelling and skin thickening of the left breast. No drainable fluid collections were identified (Fig. 5). Her symptoms were resolved following the completion of the antibiotic course.

## Discussion

This case report highlights a significant clinical challenge: differentiating between breast abscess and breast malignancy, particularly IBC, which can present with overlapping features [[8], [9], [10]]. Our patient, a 52-year-old woman with multiple comorbidities, initially presented with symptoms and imaging findings highly suggestive of breast cancer. However, subsequent diagnostic workup confirmed the presence of a breast abscess.

In this case, the mammography and US findings of an irregular, complex cystic and solid mass with associated nipple retraction were highly suggestive of IBC, which demonstrates the importance of careful differential diagnosis [9,11,12]. The patient's lack of pain and tenderness at initial presentation, alongside the normal mammography from a year prior, initially pointed away from an infectious etiology. However, the final diagnosis was made through histopathological examination of the biopsy. Her clinical progression with symptoms such as swelling, erythema, and discharge following the biopsy, combined with elevated inflammatory markers, provided clues towards an infectious process [3].

Several factors may contribute to the misdiagnosis of breast abscess as breast cancer. Nonpuerperal abscesses, often present with risk factors such as obesity, diabetes, and smoking, all of which were present in our patient [6,7]. The presence of uncontrolled diabetes and smoking likely compounded her risk for nonpuerperal abscesses and could have influenced the clinical presentation and imaging findings [13,14].

Management strategies differ significantly between breast abscesses and IBC. The initial treatment of breast abscesses typically involves antibiotics and, if necessary, drainage [3,5,15]. Our patient's initial treatment with cephalexin was inadequate due to the abscess's complexity, necessitating a switch to intravenous antibiotics and more targeted management. The absence of a drainable fluid collection on repeats US also emphasized the need for continued evaluation and appropriate adaptation of treatment plans based on patient response [4,16].

## Conclusion

In conclusion, this case highlights the diagnostic challenge between breast abscess and IBC due to overlapping clinical presentations. Initially suspected as IBC given the patient's history and imaging findings, pathological analysis confirmed a breast abscess. This case highlights the importance of meticulous evaluation combining clinical history, physical examination, imaging modalities such as mammography and US, and histopathological analysis for accurate diagnosis. Timely differentiation between infectious processes and malignancy is critical to guide appropriate management strategies and optimize patient outcomes.

## Patient consent

Written informed consent for the publication of this case report was obtained from the patient.
